# IGFBP-6/sonic hedgehog/TLR4 signalling axis drives bone marrow fibrotic transformation in primary myelofibrosis

**DOI:** 10.18632/aging.203779

**Published:** 2021-12-14

**Authors:** Lucia Longhitano, Daniele Tibullo, Nunzio Vicario, Cesarina Giallongo, Enrico La Spina, Alessandra Romano, Sofia Lombardo, Marina Moretti, Francesco Masia, Anna Rita Daniela Coda, Santina Venuto, Paolo Fontana, Rosalba Parenti, Giovanni Li Volti, Michelino Di Rosa, Giuseppe A. Palumbo, Arcangelo Liso

**Affiliations:** 1 Department of Biomedical and Biotechnological Sciences, University of Catania, Catania 95123, Italy; 2 Department of Scienze Mediche Chirurgiche e Tecnologie Avanzate “G.F. Ingrassia”, University of Catania, Catania 95123, Italy; 3 Division of Hematology, Department of General Surgery and Medical-Surgical Specialties, A.O.U. “Policlinico-Vittorio Emanuele”, University of Catania, Catania 95123, Italy; 4 Department of Medical Oncology, The Mediterranean Institute of Oncology, Viagrande 95029, Italy; 5 Department of Medicine, University of Perugia, Perugia 06129, Italy; 6 Department of Medical and Surgical Sciences, University of Foggia, Foggia 71100, Italy

**Keywords:** IGFBP-6, myelofibrosis, mesenchymal stem cells, TLR4, MPNs

## Abstract

Primary myelofibrosis is a Ph-negative chronic myeloproliferative neoplasm characterized by bone marrow fibrosis and associated with the involvement of several pathways, in addition to bone marrow microenvironment alterations, mostly driven by the activation of the cytokine receptor/JAK2 pathway. Identification of driver mutations has led to the development of targeted therapy for myelofibrosis, contributing to reducing inflammation, although this currently does not translate into bone marrow fibrosis remission. Therefore, understanding the clear molecular cut underlying this pathology is now necessary to improve the clinical outcome of patients. The present study aims to investigate the involvement of IGFBP-6/sonic hedgehog /Toll-like receptor 4 axis in the microenvironment alterations of primary myelofibrosis. We observed a significant increase in IGFBP-6 expression levels in primary myelofibrosis patients, coupled with a reduction to near-normal levels in primary myelofibrosis patients with JAK2V617F mutation. We also found that both IGFBP-6 and purmorphamine, a SHH activator, were able to induce mesenchymal stromal cells differentiation with an up-regulation of cancer-associated fibroblasts markers. Furthermore, TLR4 signaling was also activated after IGFBP-6 and purmorphamine exposure and reverted by cyclopamine exposure, an inhibitor of the SHH pathway, confirming that SHH is involved in TLR4 activation and microenvironment alterations. In conclusion, our results suggest that the IGFBP-6/SHH/TLR4 axis is implicated in alterations of the primary myelofibrosis microenvironment and that IGFBP-6 may play a central role in activating SHH pathway during the fibrotic process.

## INTRODUCTION

A large number of hematologic and non-hematologic disorders are associated with increased bone marrow (BM) fibrosis, an abnormal accumulation of extracellular matrix (ECM) components, representing the endpoint of many chronic inflammatory diseases, leading to organ dysfunction [1]. Primary myelofibrosis (PMF) is an hematologic disease characterized by progressive proliferation of mainly granulocytic and megakaryocytic cells in the bone marrow, which in turn stimulate BM fibrosis, eventually resulting in extramedullary hematopoiesis and massive splenomegaly [2, 3]. Patients with PMF display aberrant expression of several cytokines, implicated as either the cause or the effect of the bone marrow stromal reaction [4]. PMF is still considered an incurable disease, with the notable exception of the few patients who successfully undergo an allogeneic stem cell transplantation [5]. The molecular pathogenesis of PMF is characterized by dysregulation of the Janus kinase/STAT (JAK/STAT) signalling pathway, which is crucial for normal cytokine-mediated cell responses. Unfortunately, up to date, the only available target therapy is represented by the JAK inhibitors (JAKi), which can reduce the spleen size and relieve constitutional symptoms by acting on the inflammatory cascade. However, JAKi are neither able to restore the altered stroma nor to revert bone marrow fibrosis [6]. Another important pathway, frequently involved in PMF and secondary myelofibrosis (SMF), is the Hedgehog signalling pathway, also recognized in several fibrotic and malignant diseases. Increased expression of Hedgehog target genes was reported in granulocytes isolated from unselected MPN patients, while activation of Hedgehog signalling was reported to be present in murine BM transplant models of PMF [7]. Among the Hedgehogs, sonic hedgehog (SHH) is the best characterized and most widely studied. SHH signalling has been implicated in the regulation of injury repair and its expression is deregulated in a multitude of fibrotic processes [8–10]. Consequently, targeting SHH signalling might be a promising strategy for therapeutic intervention in a multiplicity of fibrotic diseases [11]. The pathogenesis of fibrosis is not fully understood [12], but several lines of evidence suggest that various cytokines or growth factors [13] may promote a supporting microenvironment, leading to the development of a profibrotic population of fibroblasts. A regulatory loop between chemokines and transforming growth factor-beta (TGFβ) has been described [14, 15]. In particular, ECM components store TGFβ which remains as a latent signal until activation via MMP-dependent proteolysis or by mechanical tension [16]. After activation, TGFβ favors mesenchymal stromal cells mediated ECM deposition through induction of type I, III, IV, VII, and X collagen, fibronectin, and proteoglycans [17]. The critical role of TGFβ has been established in a variety of fibrotic disorders relating to the severity of fibrosis and a low count of BM megakaryocytes [18, 19]. It has been demonstrated that TGF-β produced by hematopoietic cells is pivotal for the pathogenesis of myelofibrosis that develops in mice with thrombopoietin overexpression [20–22]. Few effective therapies to stop or reverse tissue fibrosis are available in clinical practice. Thus, it is important to understand the cellular and molecular mechanisms of fibrogenesis, to fully understand the pathogenesis of the fibrotic process, and also to develop efficient strategies to treat patients with fibrotic disorders [11].

Insulin-like growth factor-binding proteins (IGFBPs) play a relevant role in the fibrotic process in the liver damage [23]. In particular, it has been demonstrated a mutual regulation between IGFBP-7 and TGF-β in hepatic stellate cells which most likely accelerates the progression of liver fibrosis [24]. Interestingly, patients with PMF display significantly higher levels of insulin-like growth factor-binding protein-2 (IGFBP-2), confirming the pathogenetic role of these proteins in PMF progression [4]. Interestingly, higher levels of IGFBP-2 have been described in patients with PMF compared to healthy donors [4], suggesting a potential pathogenetic role of IGFBPs in PMF. Given the role of IGFBP-6 as a master regulator of fibroblasts proliferation and senescence [25–28], in the current study, we analyzed its role in the molecular pathogenesis of PMF also to further elucidate the molecular mechanism(s) of PMF fibrosis.

## MATERIALS AND METHODS

### Cell culture and pharmacological treatments

Healthy mesenchymal stem cells HS5 were purchased from ATCC Company (Milan, Italy). Cells were maintained in culture medium (Dulbecco's Modified Eagle Medium (DMEM) containing 10% fetal bovine serum (FBS), 100 U/mL penicillin, and 100 U/mL streptomycin). At 80% confluency, cells were passaged using trypsin-EDTA solution (0.05% trypsin and 0.02% EDTA) [29]. IGFBP-6 (Sigma–Aldrich, Milan, Italy) was added to the cell cultures of all experiments at final concentrations of 200 ng/mL for both 24h and 48h.

### Isolation of high-density neutrophils

Whole blood (40 mL) was collected from healthy volunteers in vacutainer tubes containing the anticoagulant, potassium EDTA, and diluted 1:1 with dextran 3% for two hours to obtain plasma enriched with white cells. Peripheral blood mononuclear cells (PBMCs) were then isolated using Ficoll-Paque, according to the manufacturer’s instructions (Pharmacia LKB Biotechnology, NJ, USA). The pellet obtained after centrifugation of PB on Ficoll, containing erythrocytes and neutrophils, was subjected to hypotonic lysis (155 mM NH^4^Cl, 10 mM KHCO^3^, 0.1 mM EDTA, pH 7.4) for 15 min on ice. Following washes, cells were sorted using the EasySep human neutrophil Isolation kit (StemCell Technology, cat #17957). Neutrophil’s purity and viability were checked by morphology and flow cytometry.

### Datasets selection

The NCBI Gene Expression Omnibus (GEO) database (http://www.ncbi.nlm.nih.gov/geo/) [30] was used to select transcriptome datasets to analyze genes expression in primary myelofibrosis (PMF) patients. Potential interesting datasets were identified using the mesh terms “myelofibrosis”, “JAK2^V617F^”, “JAK2”, “CD34^+^” and “Human”. The obtained datasets were ranked by number of samples (High to Low), age, gender, and clinical data made available by the authors. We selected two datasets (GSE53482, GSE41812,). A total of 78 samples (62 PMF patients and 16 healthy controls) were analyzed. The collection of the data samples is reported in Table 1. Supplementary information of sample recruited are available in Series Matrix File (s) (GEODataset).

**Table 1:** Dataset information.

**N°**	**Dataset**	**GPL**	**IGFBP-6**	**IDH1**	**Healthy**	**PMF**	**CD34^+^**	**JAK2^V617F^**	**^+^JAK2^V617F -^**
1	GSE53482	GPL13667	11717909_at	11718474_a_at	16	42	PB	23	19
2	GSE41812	GPL13667	11717909_at	11718474_a_at	0	20	PB	11	9

The GSE53482 (platform GPL13667) was composed of Peripheral Blood (PB) CD34^+^ Cells from 16 healthy donors and 42 PMF patients (23 PMF patients carrying the mutation JAK2^V617F^ and 19 JAK2 wild-type samples) [31]. We selected data from GSE41812 (platform GPL13667) relative to PB CD34^+^ cells of 20 PMF patients (11 carrying the mutation JAK2^V617F^ and 9 were wild-type) [32].

### Dataset processing, experimental design, and statistic

To process and identify Significantly Different Expressed Genes (SDEG) in all the selected datasets, we used the MultiExperiment Viewer (MeV) software. For multiple genes probes that insisted on the same GeneID, the ones with the highest variance were selected. For all data sets, the significance threshold level was p<0.05. The genes with p<0.05 were identified as SDEG and selected for further analysis. For all datasets, we performed statistical analysis with GEO2R, applying a Benjamini and Hochberg (False discovery rate) to adjust P values for multiple comparisons [33–35].

From all datasets, we performed a comparative analysis of significantly expressed genes in PB CD34^+^ Cells from PMF patients carrying the mutation JAK2^V617F^ compared to JAK2 wild-type PMF patients. We obtained 1278 upregulated and 2070 downregulated genes in JAK2^V617F^ mutated patients compared to JAK2 wild-type.

For statistical analysis, Prism 8 software (GraphPad Software, USA) was used. Based on the Shapiro-Wilk test, almost all data were skewed, so parametric tests were used. Significant differences between groups were assessed using the Mann–Whitney U test, and the Kruskal-Wallis test was performed to compare data between all groups followed by Dunn's post hoc test. Correlations were determined using Pearson’s ρ correlation. All tests were two-sided and significance was determined at p < 0.05. The analysis of microarray data by Z-score transformation was used to allow the comparison of microarray data independently of the original hybridization intensities [36]. Raw intensity data for each experiment is log10 transformed and then used for the calculation of Z scores. Z scores are calculated by subtracting the overall average gene intensity (within a single experiment) from the raw intensity data for each gene, and dividing that result by the SD of all of the measured intensities, according to the formula:

Z score (intensity G − mean intensity G1. . . Gn)/SDG1. . . Gn

where G is any gene on the microarray and G1. . . Gn represents the aggregate measure of all the genes [37].

### Real-time PCR

RNA was extracted by Trizol® reagent (category no. 15596026, Invitrogen, Carlsbad, CA, USA). The first-strand cDNA was then synthesized with a High-Capacity cDNA Reverse Transcription kit (category no. 4368814, Applied Biosystems, Foster City, CA, USA). High cDNA quality was checked, taking into consideration the housekeeping gene Ct values. Quantitative real-time PCR was performed in Step-One Fast Real-Time PCR system, Applied Biosystems, using the SYBR Green PCR MasterMix (category no. 4309155, Life Technologies, Monza, Italy). The specific PCR products were detected by the fluorescence of SYBR Green, the double-stranded DNA binding dye. Primers were designed using BLAST® (Basic Local Alignment Search Tool, NCBI, NIH), considering the shortest amplicon proposed and β-actin was used as the housekeeping gene. Primers were purchased by Metabion International AG (Planneg, Germany) (Table 2). The relative mRNA expression level was calculated by the threshold cycle (Ct) value of each PCR product and normalized with β-actin by using a comparative 2-ΔΔCt method.

**Table 2:** Gene of interest primer sets.

**Gene of interest**	**Forward primer (5′ → 3′)**	**Reverse primer (5′ → 3′)**
TGFB	CCCAGCATCTGCAAAGCTC	GTCAATGTACAGCTGCCGCA
BMP2	ATGGATTCGTGGTGGAAGTG	GTGGAGTTCAGATGATCAGC
SHH	GCGAGATGTCTGCTGCTAGT	TTACACCTCTGAGTCTCAGCC
IGFBP-6	GACCAGGAAAGAATGTGAAAGGA	GCTCTGCCAATTGACTTTCCTTAG
β-Actin	CCTTTGCCGATCCGCCG	AACATGATCTGGGTCATCTTCTCGC

### Western blot analysis

Briefly, for western blot analysis, 50 μg of proteins were loaded onto a 12% polyacrylamide gel Mini-PROTEAN® TGXTM (BIO-RAD, Milan, Italy). Electro-transfer to nitrocellulose membrane was obtained through Trans- Blot® TurboTM (BIO-RAD), using Trans-Blot® SE Semi-Dry Transfer Cell (BIO-RAD). Membranes were blocked in Odyssey Blocking Buffer (Licor, Milan, Italy), according to the manufacturer’s protocol. After blocking, membranes were washed three times in PBS for 5 min and incubated with primary antibodies against human α-SMA, TGF-β, HMOX1, TLR3, TLR4, E – Cadherin, Col1a, and β-actin (Santa Cruz Biotechnology, Santa Cruz, CA, USA), overnight at 4° C. The next day, membranes were washed three times in PBS for 5 min and incubated with Infrared anti-mouse IRDye800CW (1:5000) and anti-rabbit IRDye700CW secondary antibodies (1:5000) in PBS/0.5% Tween-20 for 1h at room temperature. All the antibodies were diluted in Odyssey Blocking Buffer. The obtained blots were visualized by Odyssey Infrared Imaging Scanner (Licor, Milan, Italy). Densitometric analysis was used for protein levels quantification, normalizing data to protein levels of β-actin.

### Cytokine detection

Cell culture supernatants collected 24h from cell-laden hydrogel were frozen at −80° C until use. Multiplex immunobead assay technology (procartaplex Cytokine/Chemokine Magnetic Bead Panel, THERMO, MA; and Magpix analytical test instrument, Luminex Corp., Austin, TX) was performed on culture medium to determine concentrations of selected cytokines (BMP2, IL – 6, MMP2, CHI3L1, RANTES/CCL5, sRANKL, OPG, MMP9, TIMP – 1, IL – 8, MCP - 1). Culture medium from untreated cells and treated were evaluated.

### Immunocytochemistry analysis

Cells were grown directly on coverslips before immunofluorescence and treated with IGFBP-6 at the final concentration of 200 ng/mL. After washing with PBS, cells were fixed in 4% paraformaldehyde (category no. 1004968350 Sigma-Aldrich, Milan, Italy) for 20min at room temperature. Subsequently, cells were incubated with primary antibody against p – NFkB, IRF3, and YAP1 at 1:200 dilution, overnight at 4° C. After 24h, cells were washed three times in PBS for 5 min and then incubated with TRITC secondary antibody (anti-goat, Santa Cruz Biotechnology, Santa Cruz, CA, USA) (1:200) for 1h at room temperature. The slides were mounted with a medium containing DAPI (4′,6- diamidino-2phenylindole, category no. sc-3598, Santa Cruz Biotechnology, Santa Cruz, CA, USA) to visualize nuclei. The fluorescent images were obtained using a Zeiss Axio Imager Z1 microscope with Apotome 2 system (Zeiss, Milan, Italy). The specificity of immunostaining was verified by excluding incubation with the primary or secondary antibody, as the negative control. Immunoreactivity was evaluated considering the signal-to-noise ratio of immunofluorescence [38].

### Statistical analysis

Statistical analyses were performed using SPSS11.0 software. Differences between experimental groups were determined by the Fisher method with statistical significance (p<0.05). To compare treatment groups, the null hypothesis was tested by single-factor analysis of variance (ANOVA) for multiple groups. Likewise, the unpaired T-test method was used for two groups. Data are presented as mean ± SD.

## RESULTS

### IGFBP-6 was modulated in PMF patients

**Figure 1:**
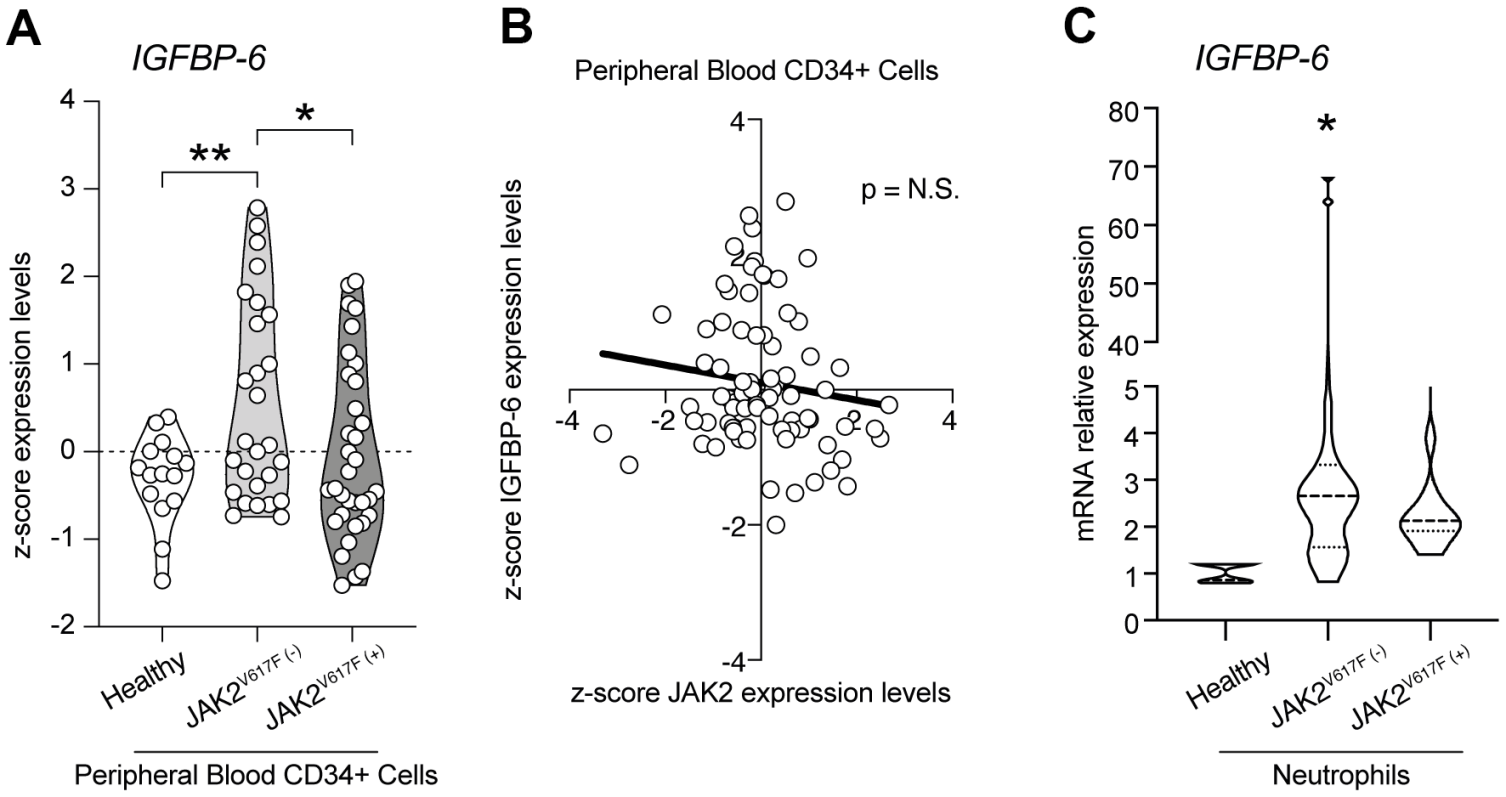
**IGFBP-6 was modulated in PMF patients.** (**A**) z-score expression levels of IGFBP-6 in healthy, JAK2 wild type, and JAK2V617F mutant PMF patients. (**B**) Correlation analysis between IGFBP-6 and JAK2 expression levels in the PMF patients. (**C**) mRNA expression of IGFBP-6 in neutrophils of PMF patients. * p<0.05.

To find a potential link between JAK2 and IGFBP-6, we first analyzed the z-score expression levels of IGFBP-6 in healthy, JAK2 wild type and JAK2V617F mutant PMF patients (Figure 1A). We observed a significant increase in IGFBP-6 z-score gene expression levels in PMF patients wild type for JAK2V617F mutation compared to healthy and to PMF patients who carried the JAK2V617F mutation (Figure 1A). Furthermore, no significant modulation was observed comparing IGFBP-6 expression levels between healthy subjects and PMF patients who carried the JAK2V617F mutation. Noteworthy, we observed no significant correlation between IGFBP-6 and JAK2 expression levels in the selected samples for our analysis (Figure 1B). We then moved to analyze IGFBP-6 mRNA expression in primary isolated neutrophils positive and negative cells from PMF patients (Figure 1C). Interestingly, we observed that also in PMF JAK2^WT^neutrophils, IGFBP-6 mRNA expression was significantly increased.

### IGFBP-6 induces CAF differentiation in mesenchymal stem cells

**Figure 2:**
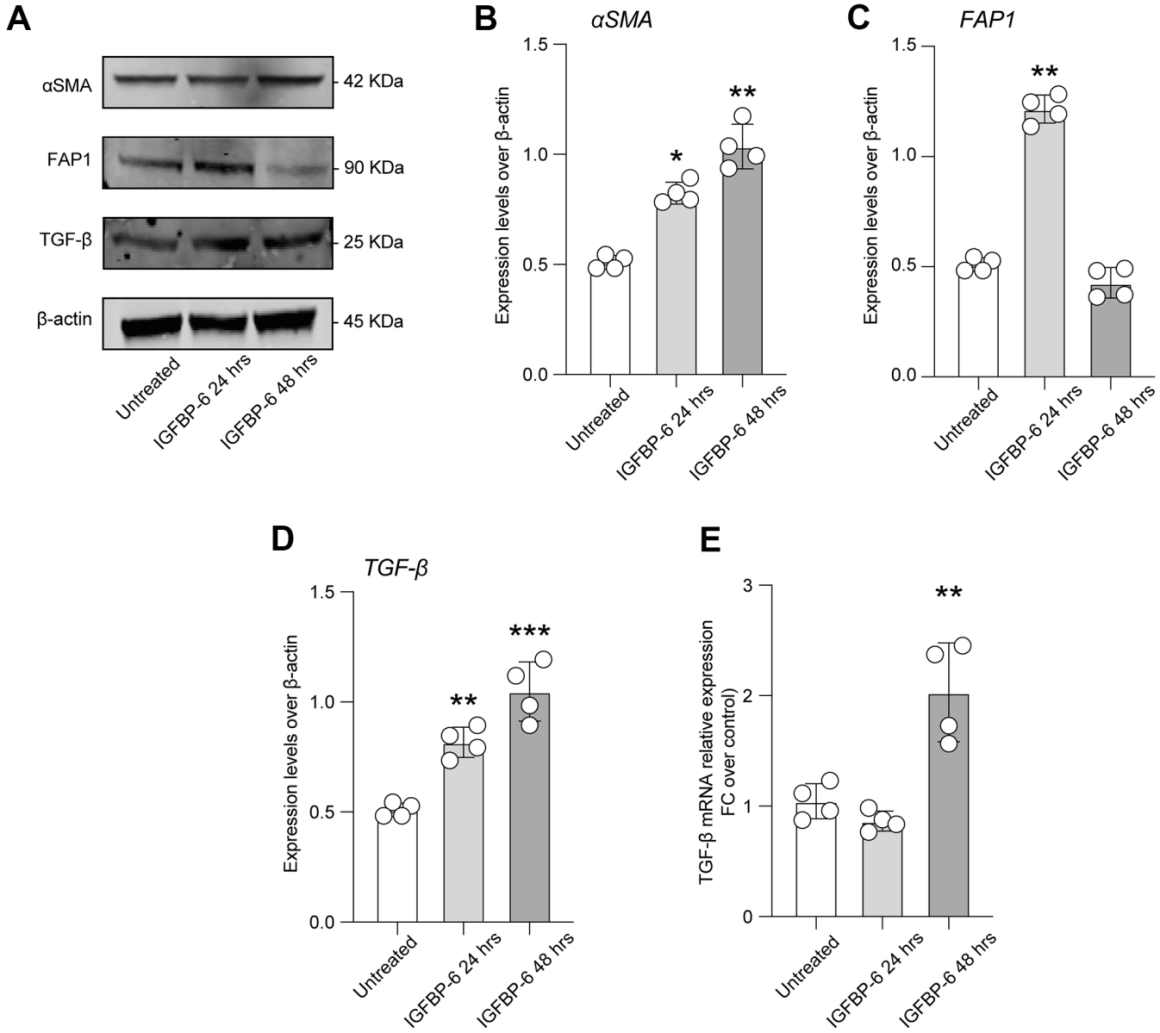
**IGFBP-6 induces CAF differentiation in mesenchymal stem cells.** (**A**) HS5 cells exposed to 200 ng/mL of IGFBP-6 for 24h and 48h were lysed and subjected to immunoblotting using specific antibodies against α-SMA, FAP1, and TGF-β. Protein content was normalized to the housekeeping protein β-actin. The entire assay was made in triplicate, a representative one is shown. Signals from immunodetected bands were semi-quantified by densitometry. (**B**–**D**) Statistical analysis of data revealed that α-SMA expression was significantly increased in the HS5 cells IGFBP-6- induced for 24h and 48h (**B**), FAP1 expression was significantly increased in the HS5 cells IGFBP-6- induced for 24h (**C**) TGF-β expression was significantly increased in the HS5 cells IGFBP-6- induced for 24h and 48h (**D**). Data are presented as means ± sem. **p < 0.01 and ***p < 0.001 vs. untreated. (**E**) qPCR results were obtained for TGF-β in HS5 cells exposed to 200 ng/mL of IGFBP-6 for 24h and 48h. Relative mRNA expression level normalized with β-actin by using a comparative 2-ΔΔCt method. **p < 0.01.

In order to highlight cellular mechanisms involved in BM fibrosis, we moved to study the effects of IGFBP-6 signalling in HS5 cells. We exposed HS5 cells to 200 ng/mL of IGFBP-6 for 24h and 48h and we then analyzed the expression levels of cancer-associated fibroblasts (CAFs) markers. Western blot analysis revealed that 24h post-IGFBP-6 exposition, the expression levels of the CAFs markers α-smooth muscle actin (α-SMA, Figure 2A, 2B), fibroblast activation protein (FAP, Figure 2A, 2C), and TGF-β (Figure 2A, 2D) were significantly increased as compared to control cells (Figure 2A–2D), indicating a CAFs transition of HS5 cells upon IGFBP-6 signalling stimulation. α-SMA and TGF-β levels were significantly upregulated also 48h post-IGFBP-6 exposition (Figure 2A–2D) and qRT-PCR confirmed TGF-β mRNA levels at 48h as compared to control cultures (Figure 2E).

### IGFBP-6 induces genes associated with extracellular matrix and bone remodeling

To further characterize HS5 cells phenotype upon IGFBP-6 stimulation and the potential microenvironmental conditioning mediated by CAFs differentiation, we analyzed the expression levels of several tumor invasiveness and progression mediators.

**Figure 3:**
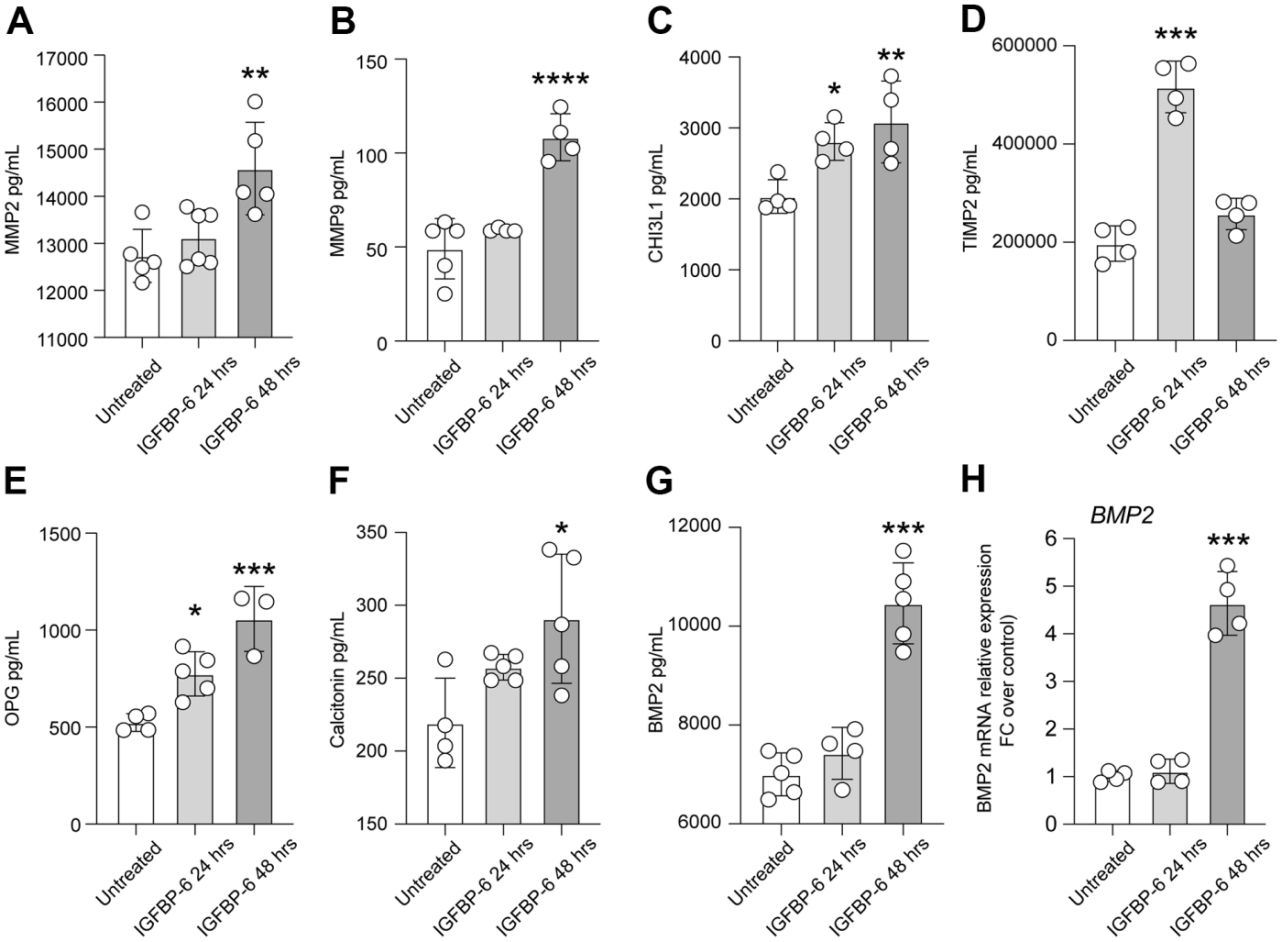
**IGFBP-6 induces the expression of mediators involved in proliferation and migration in HS5 cells.** (**A**–**G**) Multiplex immunobead assay technology on HS5 cells exposed to 200 ng/mL of IGFBP-6 for 24h and 48h was performed on a culture medium to determine concentrations of indicated cytokines. Culture medium from untreated cells and treated cells were evaluated. (*P < 0.05) (**H**) qPCR results obtained for BMP2 in HS5 cells exposed to 200 ng/mL of IGFBP-6 for 24h and 48h. Relative mRNA expression level normalized with β-actin by using a comparative 2-ΔΔCt method. *** P < 0.001.

We observed that both matrix metalloproteinase-2 (MMP2) and MMP9 were increased 48h after IGFBP-6 exposition (Figure 3A, 3B), and this was coupled with an increased expression of chitinase 3 like 1 (CHI3L1) after both 24h and 48h post-treatment (Figure 3C). We also observed that TIMP metallopeptidase inhibitor 2 (TIMP2) levels were increased by IGFBP-6 stimulation at 24h, even if our data report near-normal levels at 48h \post-IGFBP-6 exposition (Figure 3D). Also, the levels of TNF receptor superfamily member 11b osteoprotegerin (OPG) and calcitonin were found to increase over time upon IGFBP-6 exposition (Figure 3E, 3F). Finally, bone morphogenetic protein 2 (BMP2) expression was 2-fold increased in IGFBP-6 treated cells as compared to control (Figure 3G) and this was supported by an increased BMP2 mRNA expression (Figure 3H).

### Purmorphamine-mediated SMO activation recapitulates IGFBP-6 effects on HS5 cells

**Figure 4:**
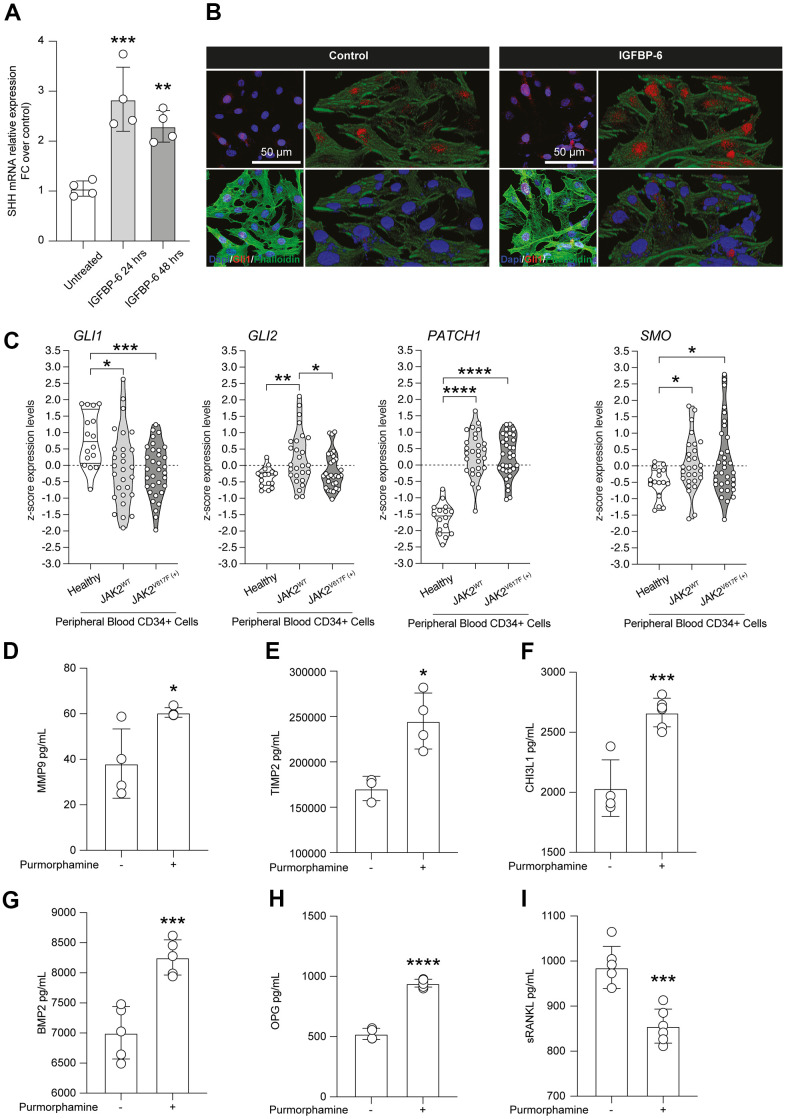
**Purmorphamine-mediated SMO activation recapitulates IGFBP-6 effects on HS5 cells.** (**A**) qPCR results were obtained for SHH in HS5 cells exposed to 200 ng/mL of IGFBP-6 for 24h and 48h. Relative mRNA expression level normalized with β-actin by using a comparative 2-ΔΔCt method. **p <0.01; ***p < 0.001. (**B**) Immunofluorescence analysis were performed on HS5 cells treated with IGFBP-6 at the final concentration of 200 ng/mL, followed by fixing and staining with anti-Phalloidin (green) and anti-Gli1 (red). Nuclei were visualized using DAPI. Immunoreactivity was evaluated considering the signal-to-noise ratio of immunofluorescence (scale bar 20 μm). (**C**) z-score expression levels of Gli1, Gli2, GPATCH1, and SMO in healthy, JAK2 wild type, and JAK2V617F mutant PMF patients. (**D**–**I**) Multiplex immunobead assay technology on HS5 cells exposed or not to purmorphamine was performed on culture medium to determine concentrations of indicated cytokines. Histograms showed a significant increase of MMP9 (**D**), TIMP (**E**), CHI3L1 (**F**), BMP2 (**G**), OPG (**H**), and sRANKL (**I**) after purmorphamine stimulation, as compared to control. **p <0.01; ***p < 0.001.

In the effort to link IGFBP-6 effects with key inductors and regulators of cell fate and differentiation in the fibrotic process, we analyzed whether IGFBP-6 stimulation was coupled with SHH de-regulation. SHH mRNA expression levels in control and IGFBP-6 treated cells were robustly increased at both 24h and 48h post-stimulation (Figure 4A). Such a phenomenon, IGFBP-6 stimulation was also coupled with an increase of GLI family zinc finger 1 (GLI1) nuclear translocation (Figure 4B), indicating that IGFBP-6 stimulation was able to induce the canonical SHH signalling pathway on HS5 cells.

Noteworthy we analyzed SHH signalling pathway in peripheral blood-derived CD34+ cells sorted from microarray datasets and we observed that the GLI1 and GLI2 z-score expression levels in PMF patients who carried the JAK2V617F mutation were significantly reduced, compared to healthy subjects (Figure 4C). Furthermore, we highlighted a concomitant increase of the SHH receptor patched 1 (PTCH1) and the effector belonging to the pathway smoothened (SMO) in both PMF patient groups, JAK2WT and JAK2V617F mutant, compared to the healthy subjects (Figure 4C).

Given the relevance and druggability of the SHH signalling pathway, we analyzed the levels of proliferation and migration mediators found as induced by IGFBP-6, in HS5 cells exposed to the SMO agonist purmorphamine. Intriguingly, we observed an IGFBP-6 superimposable increase of MMP9 (Figure 4D), TIMP (Figure 4E), CHI3L1 (Figure 4F), BMP2 (Figure 4G), and OPG (Figure 4H) upon purmorphamine stimulation, as compared to control levels. Such evidence was coupled with a significant reduction of Soluble RANK Ligand (sRANKL) in purmorphamine-treated HS5 cells (Figure 4I).

### SHH/IGFBP-6/TLR4 axis activation in HS5 cells

**Figure 5:**
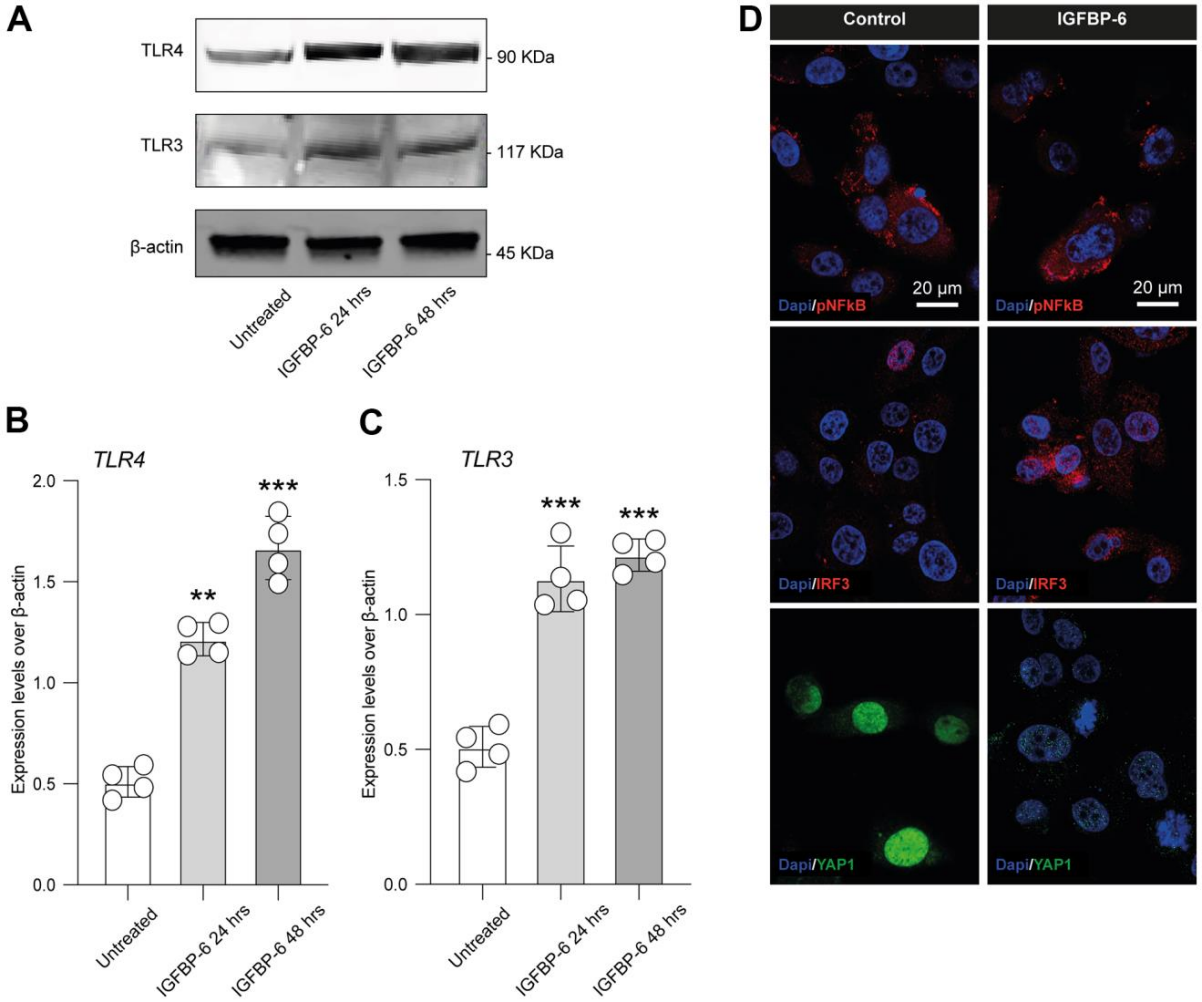
**IGFBP-6 induces TLR4 signalling on HS5 cells.** (**A**) HS5 cells exposed to 200 ng/mL of IGFBP-6 for 24h and 48h were lysed and subjected to immunoblotting using specific antibodies against TLR4 and TLR3. Protein content was normalized to the housekeeping protein β-actin. The entire assays were made in triplicate, a representative one is shown. Signals from immunodetected bands were semi-quantified by densitometry. (**B**, **C**) Statistical analysis of data revealed that TLR4 (**B**) and TLR3 (**C**) expression were significantly increased in the HS5 cells IGFBP-6- induced for 24h and 48h. Data are presented as means ± sem. **p < 0.01 and ***p < 0.001 vs. untreated. (**D**) Immunofluorescence analysis were performed on HS5 cells treated with IGFBP-6 at the final concentration of 200 ng/mL, followed by fixing and staining with anti-pNF-kB (red), anti-IRF3 (red), and anti-YAP1 (green). Nuclei were visualized using DAPI. Immunoreactivity was evaluated considering the signal-to-noise ratio of immunofluorescence (scale bar 20 μm).

Given the toll-like receptor 4 (TLR4) and TLR3 role in PMF, we moved to analyze the expression levels of TLR4 and TLR3 in HS5 cells after IGFBP-6 stimulation at both 24h and 48h. We found increased protein expression levels of both receptors as compared to control (Figure 5A–5C), indicating that IGFBP-6 was able to modulate TLR4 and TLR3 signalling.

We also performed an immunofluorescence analysis in IGFBP-6 exposed and control cells for phospho nuclear factor kappa B (pNF-kB), interferon regulatory factor 3 (IRF3), and yes-associated protein 1 (YAP1, Figure 5D), finding that IGFBP-6 exposition increased immunofluorescence intensity of IRF3 while reduced pNF-kB and suppressed nuclear YAP1 in exposed HS5 cells (Figure 5D).

**Figure 6:**
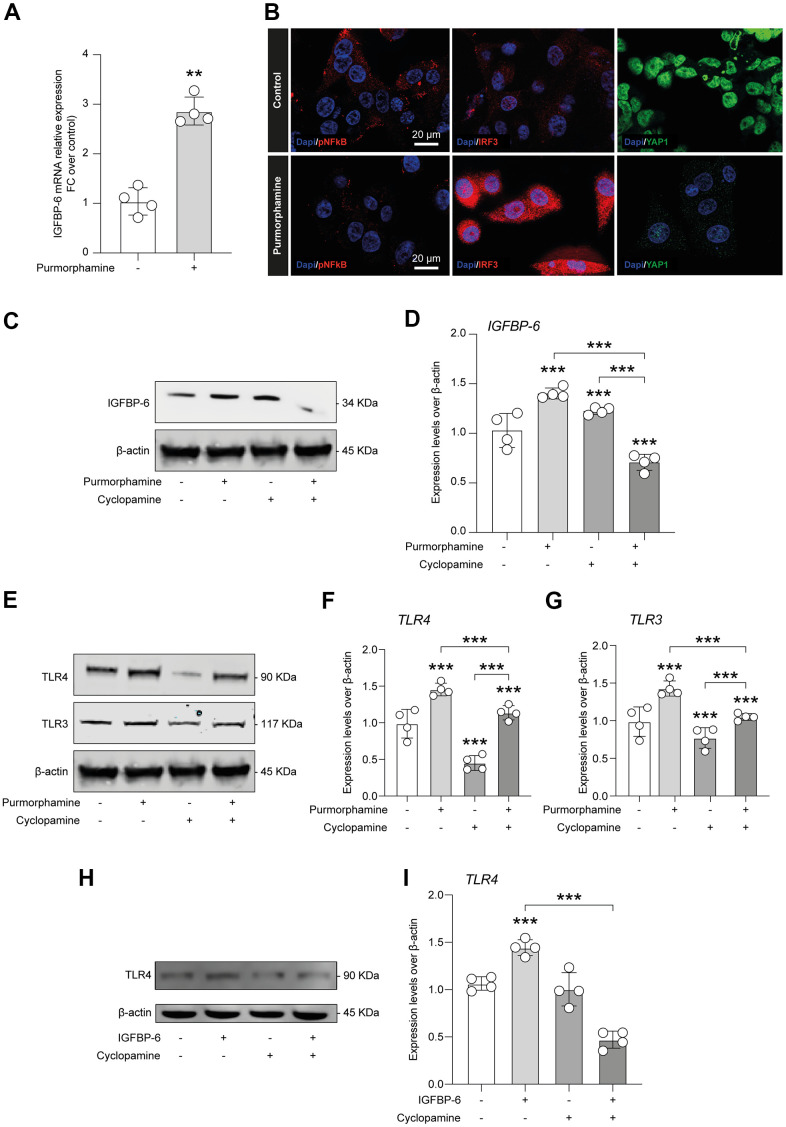
**IGFBP-6-induced TLR4 signalling is controlled by SHH signalling through SMO.** (**A**) qPCR results obtained for IGFBP-6 in HS5 cells exposed or not to purmorphamine. Relative mRNA expression level normalized with β-actin by using a comparative 2-ΔΔCt method. **P < 0.01 and ***P < 0.001. (**B**) Immunofluorescence analysis were performed on HS5 cells exposed or not to purmorphamine, followed by fixing and staining with anti-pNF-kB (red), anti-IRF3 (red), and anti-YAP1 (green). Nuclei were visualized using DAPI. Immunoreactivity was evaluated considering the signal-to-noise ratio of immunofluorescence (scale bar 20 μm). (**C**) HS5 cells exposed to purmorphamine, cyclopamine, or both were lysed and subjected to immunoblotting using a specific antibody against IGFBP-6. Protein content was normalized to the housekeeping protein β-actin. The entire assay was made in triplicate, a representative one is shown. Signals from immunodetected bands were semi-quantified by densitometry. (**D**) Statistical analysis of data revealed that IGFBP-6 expression was significantly increased after exposure to purmorphamine. Data are presented as means ± sem. **p < 0.01 and ***p < 0.001 vs. untreated. (**E**) HS5 cells exposed to purmorphamine, cyclopamine, or both were lysed and subjected to immunoblotting using specific antibodies against TLR4 and TLR3. Protein content was normalized to the housekeeping protein β-actin. The entire assay was made in triplicate, a representative one is shown. Signals from immunodetected bands were semi-quantified by densitometry. (**F**, **G**) Statistical analysis of data revealed that purmorphamine was able to increase while cyclopamine was able to suppress both TLR4 (**F**) and TLR3 (**G**) protein expression levels. Co-treatment with both SMO agonist and antagonist did not affect TLR4 and TLR3 expression levels, as compared to control cell cultures. Data are presented as means ± sem. **p < 0.01 and ***p < 0.001 vs. untreated. (**H**) HS5 cells exposed to IGFBP-6, cyclopamine, or both were lysed and subjected to immunoblotting using a specific antibody against TLR4. Protein content was normalized to the housekeeping protein β-actin. The entire assay was made in triplicate, a representative one is shown. Signals from immunodetected bands were semi-quantified by densitometry. (**I**) Statistical analysis of data revealed that TLR4 expression levels were significantly increased after IGFBP-6 stimulation, while a cotreatment with cyclopamine had a reducing effect on TLR4 expression. Data are presented as means ± sem. **p < 0.01 and ***p < 0.001 vs. untreated.

In the effort to link SHH signalling stimulation throughout purmorphamine, IGFBP-6 effects, and TLR4, we first analyzed whether purmorphamine was able to increase IGFBP-6 mRNA levels, confirming a mechanistic link between SMO activation and IGFBP-6 expression levels (Figure 6A). Importantly, we also found that purmorphamine stimulation was able to induce a similar immunofluorescence profile of pNF-kB, IRF3, and YAP1 as compared to HS5 cells exposed to IGFBP-6 (Figure 5D, 6B). Indeed, we found increased IRF3 in purmorphamine stimulated cell cultures and reduced expression of pNF-kB and YAP1 (Figure 6B). We then moved to analyze protein expression levels upon positive (i.e. purmorphamine) and negative (i.e. cyclopamine) modulation of SHH signalling pathway. Evaluating the effects of purmorphamine-induced SMO activation, we found that purmorphamine was able to increase both TLR4 and TLR3 protein expression levels (Figure 6E–6G), and even more that cyclopamine, a SMO antagonist, was able to suppress TLR4 and TLR3 signalling (Figure 6E–6G). Of note, co-treatment with both SMO agonist and antagonist did not affect TLR4 and TLR3 expression levels, as compared to control cell cultures (Figure 6E–6G).

We finally quantified expression levels of TLR4 after IGFBP-6 stimulation and cyclopamine-mediated SMO inhibition, confirming that IGFBP-6 increases TLR4 levels but, importantly, this was abolished by cotreatment with the SMO antagonist cyclopamine (Figure 6H–6I).

## DISCUSSION

In this study, we showed that IGFBP-6 is modulated in PMF patients and mediates CAFs differentiation, by regulating the expression of α-smooth muscle actin (α-SMA), TGF-β and FAP-1 and also inducing the expression of critical factors for cell proliferation and migration in a stromal cell line. Our data also demonstrated that IGFBP-6 acts as a SHH signalling pathway modulator, resembling purmorphamine-mediated SMO activation in HS5 cells. Finally, IGFBP-6 regulation on TLR4 and TLR3 signalling, pNF-kB, and IRF3 expression, and YAP1 subcellular localization, provide novel and relevant insights on an emerging role for IGFBP-6 in controlling the fibrotic process, with implications in fibrosis pathogenesis in PMF patients.

IGFBP-6 has never been reported as related to PMF, even though increased levels of IGF-2 and IGFBP-1, IGFBP-2, IGFBP-4 transcripts have previously been found in patients with PMF [4]. Interestingly, our study shows an increased IGFBP-6 level in PMF patients with wild-type JAK2, while IGFBP-6 was reduced in PMF patients bearing JAK2^V617F^ mutation.

IGFs are involved in fibroblast activation and mobilization and can be considered key chemotactic factors, playing a crucial role in promoting stromal fibroblast transition to CAFs [28]. Consistently, silencing IGFBP-6, IGF-I, or IGF-II expression in epithelial cells or blocking IGF-1 receptor (IGF-1R) activity in fibroblasts significantly inhibits fibroblast mobilization [28]. Our data show that the exposure of HS5 cells to a recombinant IGFBP-6 protein increases the expression of CAFs specific markers α-SMA, fibroblast activation protein (FAP1), and TGF-β1 [28, 39]. Interestingly, TGF-β is a multifunctional protein associated with lung fibrosis and tumor invasion. Indeed, cancer cells might modify the bone marrow niche by releasing fibrotic and angiogenic cytokines, including TGF-β1. TGF-β is also implicated in the pathogenesis of PMF and PMF development is attributable to aberrant interactions between neoplastic hematopoietic clones and mesenchymal stromal cells (MSCs) [40]. We also observed that the expressions of α-SMA and FAP1 gradually increased in a time-dependent manner, suggesting that they were upregulated following the rise of TGFβ1 expression in HS5 cells exposed to recombinant IGFBP-6. Indeed, our data indicated that α-SMA and TGF-β1 were still up-regulated after 48h after IGFBP-6 exposition and qRT-PCR confirms a significant increase of TGF-β mRNA levels also after 48h. Therefore, it can be assumed that fibroblasts activities are gradually enhanced during the initial stage of new tissue formation and IGFBP-6 may promote CAFs formation via regulating the fibroblast functions.

Hallmarks of PMF include expansion of the megakaryocytic (MKs) lineage and bone marrow fibrosis with a progressive deposition of ECM components in the BM that favor the aberrant MKs differentiation. Our data showed that IGFBP-6 is able to induce high levels of MMP2 and MMP9, and tissue inhibitors of metalloproteinase (TIMP). Interestingly, it has been recently reported that IGFBP-7 participates in MMP2/TIMP2 and MMP9/TIMP1 balance, down-regulation of TGFβ1 expression and degradation of the ECM. Moreover, through the regulation of MMPs/TIMPs balance, SHH pathway mediates IGFBP-7 knockdown-induced attenuation of hepatic fibrosis [41]. Several studies showed that marked up-regulation of TIMP-1 in PMF, as opposed to MMPs, may increase the proliferation of bone marrow fibroblasts, suggesting their functional relevance in this disease. We observed that TIMP2 levels are increased by IGFBP-6 stimulation at 24h and that TIMP2 levels return to near-normal values within 48h post-IGFBP-6 exposition, supporting the hypothesis that IGFBP-6 may modulate the BM niche, favor ECM deposition, and then MKs differentiation [4, 42–44]. PMF is also characterized by osteosclerotic tissue representing a pathological event distinguished by increased bone density and abnormal hardening and its pathogenesis is still largely unknown [45]. Our data demonstrated that IGFBP-6 increases the levels of OPG, calcitonin, and BMP2, suggesting a possible role of IGFBP-6 as a pro-osteosclerotic agent in PMF. We also showed that IGFBP-6 treatment resulted in a significant increase of non-enzymatic chitinase-3 like-protein-1 (CHI3L1), which is involved in inflammation, fibrosis progression, tissue injury and repair, and remodelling responses [46, 47]. CHI3L1 is overexpressed in different human cancers [48] and CHI3L1 levels in PMF serum are increased, indicating that it is associated with disease progression from early-stage disease (ET, PV) to the myelofibrotic stage [49]. An increasing number of studies indicated that SHH signalling pathway is recognized in several fibrotic and malignant diseases [7], including PMF [50], and murine glioma-associated oncogene 1 (GLI1), a positive effector of canonical SHH signalling pathway, supports the development of BM fibrosis, a process that seems to be responsive to specific GLI inhibition [51]. Interestingly, it was found that IGFBP-related protein 1 (IGFBPrP1) leads to HSC activation and ECM synthesis, and promotes the development of liver fibrosis via the SHH pathway activation [52, 53]. However, the mechanisms by which IGFBPrP1, MMP/TIMP balance, and the SHH signalling pathway regulate and interact with each other are not completely elucidated [41]. Our results indicated a superimposable biological effect of IGFBP-6 and purmorphamine, a purine derivative acting as selective SMO agonist and able to modulate osteoblast differentiation [54]. In particular, the robust increase of the SHH mRNA at both 24h and 48h post-IGFBP-6 stimulation, coupled with the increase of nuclear GLI family zinc finger 1 (GLI1) translocation in HS5 cells, suggest that IGFBP-6 may play a central role in activating this critical pathway for fibrosis.

Given that PMF originates in the pluripotent hematopoietic stem cell (HSC) [55], we also examined the actions of SHH effectors on CD34+ peripheral blood cells, finding a reduction of GLI1 and GLI2. Evidence show that in approximately 10% of patients with JAK2^V617F^-negative PMF, some additional regulations may occur [55]. Therefore, we hypothesized that the control of IGFBP-6 on GLI1 could match a possible role for IGFBP-6 in the genesis of PMF by the regulation of SHH pathway. To further confirm this hypothesis, we analyzed other molecules with a role in PMF pathogenesis. MSC cells express several TLRs involved in cell migration, invasion, and secretion of immune-modulating factors and can activate MSC [56]. Specifically, TLR3-primed MSCs primarily secrete immunosuppressive cytokines, whereas TLR4-primed MSC, mostly elaborate pro-inflammatory mediators [57]. Recent genetic and genomic studies associate fibrosis with TLRs and their damage-associated molecular pattern (DAMP) endogenous ligands [58]. Here we observed that IGFBP-6 was able to modulate TLRs signalling, increasing protein expression levels of both TLRs. We also showed that IGFBP-6 modulated and potentiated the action of OPG, a member of the TNF superfamily receptor, throughout its key role in inflammation and TNF-α production. To date, possible regulation of IGFBPs on the expression of TLRs has never been demonstrated, but it is known that IGF-I and IGFBP axis is effective in reducing the inflammatory response of astrocytes through decreased expression of TLR4 in gene therapy [59]. We then hypothesize that increased TLR signalling may contribute to the increase of TNF-α, through IGFBP-6 regulation, giving a new role for IGFBP-6 in this complex process.

Notably, TLR signalling pathway activation is associated with lymphoproliferative disorders, myelodysplastic syndromes and PMF [60]. One of the most TLR-4 related inflammatory protein is the interferon regulator factor 3 (IRF3), specifically involved in TLR4-induced downstream signalling [61]. IRF3 is an important YAP activator that binds YAP, leading to its nuclear retention and activation [62]. In normal fibroblasts, YAP1 is located in the cytoplasm, while in the activated cancer-associated fibroblasts, it translocates in the nucleus and promotes the expression of genes required for pro-tumorigenic functions [63]. However, little is known about the dynamics of YAP1 nuclear shuttling and there is no evidence that it is sequestered stably in the cytoplasm. Indeed, it has been found that YAP1 nuclear translocation is extensively controlled by export rate modulation [63]. Recent studies also highlighted the functional role of YAP in organ fibrosis and tumorigenesis [64]. Among the fibroblast activating molecules that can activate fibroblasts and are involved in PMF pathogenesis, we showed that IGFBP-6 raised IRF3 expression and was able to suppress the nuclear transfer of YAP1, increasing its amount in the cytoplasm.

The cytoplasmic accumulation of YAP defines the consequent loss of its transcriptional co-activator function. IGFBP-6 could therefore increase the cytoplasmic quantity of IRF3 and at the same time promote the cytoplasmic transport of YAP1, suppressing its displacement in the nucleus. Interestingly, the immunofluorescence profile induced by IGFBP-6 resembled the profile produced by purmorphamine stimulation. Indeed, purmorphamine was able to increase IGFBP-6 mRNA levels, confirming a mechanistic link between SMO activation and IGFBP-6 expression and a possible indirect regulation on IGFBP-6-mediated fibrosis. Recently, several authors showed the central role of TLR4 in PMF. During BM fibrosis progression, fibronectin EDA isoform–TLR4 axis sustains the expansions of megakaryocytes with a proinflammatory phenotype [65]. Interestingly, our data showed also that IGFBP-6 was able to exert a direct effect on the regulation of TLR4. Particularly, IGFBP-6 stimulation increased TLR4 levels but this effect was abolished by cotreatment with the SMO antagonist cyclopamine, suggesting that IGFBP-6 induced TLR4 expression through SHH pathway.

While the role of driver mutations like JAK2^V617F^ partially explains PMF pathogenesis, the functional involvement of MSC remains poorly understood [66]. Although some data are available about the role of IGFs axis on fibroblast activation and PMF pathogenesis, the role of IGFBPs and in particular of IGFBP-6 on this complex process has still been poorly explored. In conclusion, we propose a new role for IGFBP-6 in the regulation of the fibrotic process, controlling a series of inflammation modulators and fibroblast activators, suggesting that this molecule is deeply involved in the SHH signalling pathway activation. Future additional studies are now warranted to further dissect IGFBP-6 functional role in myelofibrosis.

## References

[1] Rameshwar P, Oh HS, Yook C, Gascon P, Chang VT. Substance p-fibronectin-cytokine interactions in myeloproliferative disorders with bone marrow fibrosis. Acta Haematol. 2003; 109:1–10. 10.1159/000067268 12486316

[2] Takenaka K, Shimoda K, Akashi K. Recent advances in the diagnosis and management of primary myelofibrosis. Korean J Intern Med. 2018; 33:679–90. 10.3904/kjim.2018.033 29665657 PMC6030412

[3] Mughal TI, Vaddi K, Sarlis NJ, Verstovsek S. Myelofibrosis-associated complications: pathogenesis, clinical manifestations, and effects on outcomes. Int J Gen Med. 2014; 7:89–101. 10.2147/IJGM.S51800 24501543 PMC3912063

[4] Ho CL, Lasho TL, Butterfield JH, Tefferi A. Global cytokine analysis in myeloproliferative disorders. Leuk Res. 2007; 31:1389–92. 10.1016/j.leukres.2006.12.024 17328948

[5] Longhitano L, Li Volti G, Giallongo C, Spampinato M, Barbagallo I, Di Rosa M, Romano A, Avola R, Tibullo D, Palumbo GA. The Role of Inflammation and Inflammasome in Myeloproliferative Disease. J Clin Med. 2020; 9:2334. 10.3390/jcm9082334 32707883 PMC7464195

[6] Cervantes F, Pereira A. Does ruxolitinib prolong the survival of patients with myelofibrosis? Blood. 2017; 129:832–7. 10.1182/blood-2016-11-731604 28031182

[7] Lucijanic M, Livun A, Tupek KM, Stoos-Veic T, Pejsa V, Jonjic Z, Dzankic AF, Ivic M, Kusec R. Neutral effect of Glioma-associated oncogene-1 expression on survival in myelofibrosis. Wien Klin Wochenschr. 2020; 132:464–6. 10.1007/s00508-019-01572-1 31712882

[8] Vicario N, Bernstock JD, Spitale FM, Giallongo C, Giunta MA, Li Volti G, Gulisano M, Leanza G, Tibullo D, Parenti R, Gulino R. Clobetasol Modulates Adult Neural Stem Cell Growth via Canonical Hedgehog Pathway Activation. Int J Mol Sci. 2019; 20:1991. 10.3390/ijms20081991 31018557 PMC6514872

[9] Vicario N, Spitale FM, Tibullo D, Giallongo C, Amorini AM, Scandura G, Spoto G, Saab MW, D’Aprile S, Alberghina C, Mangione R, Bernstock JD, Botta C, et al. Clobetasol promotes neuromuscular plasticity in mice after motoneuronal loss via sonic hedgehog signaling, immunomodulation and metabolic rebalancing. Cell Death Dis. 2021; 12:625. 10.1038/s41419-021-03907-1 34135312 PMC8209072

[10] Torrisi F, Alberghina C, Lo Furno D, Zappalà A, Valable S, Li Volti G, Tibullo D, Vicario N, Parenti R. Connexin 43 and Sonic Hedgehog Pathway Interplay in Glioblastoma Cell Proliferation and Migration. Biology (Basel). 2021; 10:767. 10.3390/biology10080767 34439999 PMC8389699

[11] He W, Dai C. Key Fibrogenic Signaling. Curr Pathobiol Rep. 2015; 3:183–92. 10.1007/s40139-015-0077-z 25973345 PMC4419200

[12] Kvasnicka HM, Thiele J, Bueso-Ramos CE, Sun W, Cortes J, Kantarjian HM, Verstovsek S. Long-term effects of ruxolitinib versus best available therapy on bone marrow fibrosis in patients with myelofibrosis. J Hematol Oncol. 2018; 11:42. 10.1186/s13045-018-0585-5 29544547 PMC5856218

[13] Verstovsek S, Manshouri T, Pilling D, Bueso-Ramos CE, Newberry KJ, Prijic S, Knez L, Bozinovic K, Harris DM, Spaeth EL, Post SM, Multani AS, Rampal RK, et al. Role of neoplastic monocyte-derived fibrocytes in primary myelofibrosis. J Exp Med. 2016; 213:1723–40. 10.1084/jem.20160283 27481130 PMC4995084

[14] Lu P, Takai K, Weaver VM, Werb Z. Extracellular matrix degradation and remodeling in development and disease. Cold Spring Harb Perspect Biol. 2011; 3:a005058. 10.1101/cshperspect.a005058 21917992 PMC3225943

[15] Wynn TA, Barron L. Macrophages: master regulators of inflammation and fibrosis. Semin Liver Dis. 2010; 30:245–57. 10.1055/s-0030-1255354 20665377 PMC2924662

[16] ten Dijke P, Arthur HM. Extracellular control of TGFbeta signalling in vascular development and disease. Nat Rev Mol Cell Biol. 2007; 8:857–69. 10.1038/nrm2262 17895899

[17] Winkler J, Abisoye-Ogunniyan A, Metcalf KJ, Werb Z. Concepts of extracellular matrix remodelling in tumour progression and metastasis. Nat Commun. 2020; 11:5120. 10.1038/s41467-020-18794-x 33037194 PMC7547708

[18] Johnston JB, Dalal BI, Israels SJ, Oh S, McMillan E, Begleiter A, Michaud G, Israels LG, Greenberg AH. Deposition of transforming growth factor-beta in the marrow in myelofibrosis, and the intracellular localization and secretion of TGF-beta by leukemic cells. Am J Clin Pathol. 1995; 103:574–82. 10.1093/ajcp/103.5.574 7741102

[19] Agarwal A, Morrone K, Bartenstein M, Zhao ZJ, Verma A, Goel S. Bone marrow fibrosis in primary myelofibrosis: pathogenic mechanisms and the role of TGF-β. Stem Cell Investig. 2016; 3:5. 10.3978/j.issn.2306-9759.2016.02.03 27358897 PMC4923632

[20] Chagraoui H, Komura E, Tulliez M, Giraudier S, Vainchenker W, Wendling F. Prominent role of TGF-beta 1 in thrombopoietin-induced myelofibrosis in mice. Blood. 2002; 100:3495–503. 10.1182/blood-2002-04-1133 12393681

[21] Yanagida M, Ide Y, Imai A, Toriyama M, Aoki T, Harada K, Izumi H, Uzumaki H, Kusaka M, Tokiwa T. The role of transforming growth factor-beta in PEG-rHuMGDF-induced reversible myelofibrosis in rats. Br J Haematol. 1997; 99:739–45. 10.1046/j.1365-2141.1997.4843288.x 9432016

[22] Rossi C, Zini R, Rontauroli S, Ruberti S, Prudente Z, Barbieri G, Bianchi E, Salati S, Genovese E, Bartalucci N, Guglielmelli P, Tagliafico E, Rosti V, et al, and AGIMM (AIRC-Gruppo Italiano Malattie Mieloproliferative) investigators. Role of TGF-β1/miR-382-5p/SOD2 axis in the induction of oxidative stress in CD34+ cells from primary myelofibrosis. Mol Oncol. 2018; 12:2102–23. 10.1002/1878-0261.12387 30259659 PMC6275274

[23] Rosique-Oramas D, Martínez-Castillo M, Raya A, Medina-Ávila Z, Aragón F, Limón-Castillo J, Hernández-Barragán A, Santoyo A, Montalvo-Javé E, Pérez-Hernández JL, Higuera-de la Tijera F, Torre A, Kershenobich D, Gutiérrez-Reyes G. Production of insulin-like growth factor-binding proteins during the development of hepatic fibrosis due to chronic hepatitis C. Rev Gastroenterol Mex (Engl Ed). 2020; 85:390–8. 10.1016/j.rgmx.2019.08.006 31740166

[24] Li XQ, Zhang QQ, Zhang HY, Guo XH, Fan HQ, Liu LX. Interaction between insulin-like growth factor binding protein-related protein 1 and transforming growth factor beta 1 in primary hepatic stellate cells. Hepatobiliary Pancreat Dis Int. 2017; 16:395–404. 10.1016/S1499-3872(17)60013-4 28823370

[25] Micutkova L, Diener T, Li C, Rogowska-Wrzesinska A, Mueck C, Huetter E, Weinberger B, Grubeck-Loebenstein B, Roepstorff P, Zeng R, Jansen-Duerr P. Insulin-like growth factor binding protein-6 delays replicative senescence of human fibroblasts. Mech Ageing Dev. 2011; 132:468–79. 10.1016/j.mad.2011.07.005 21820463 PMC3192261

[26] Raykha C, Crawford J, Gan BS, Fu P, Bach LA, O’Gorman DB. IGF-II and IGFBP-6 regulate cellular contractility and proliferation in Dupuytren’s disease. Biochim Biophys Acta. 2013; 1832:1511–9. 10.1016/j.bbadis.2013.04.018 23623986

[27] Xie L, Tsaprailis G, Chen QM. Proteomic identification of insulin-like growth factor-binding protein-6 induced by sublethal H2O2 stress from human diploid fibroblasts. Mol Cell Proteomics. 2005; 4:1273–83. 10.1074/mcp.M500032-MCP200 15958393

[28] De Vincenzo A, Belli S, Franco P, Telesca M, Iaccarino I, Botti G, Carriero MV, Ranson M, Stoppelli MP. Paracrine recruitment and activation of fibroblasts by c-Myc expressing breast epithelial cells through the IGFs/IGF-1R axis. Int J Cancer. 2019; 145:2827–39. 10.1002/ijc.32613 31381136

[29] Tibullo D, Barbagallo I, Giallongo C, La Cava P, Branca A, Conticello C, Stagno F, Chiarenza A, Palumbo GA, Di Raimondo F. Effects of second-generation tyrosine kinase inhibitors towards osteogenic differentiation of human mesenchymal cells of healthy donors. Hematol Oncol. 2012; 30:27–33. 10.1002/hon.988 21544849

[30] Clough E, Barrett T. The Gene Expression Omnibus Database. Methods Mol Biol. 2016; 1418:93–110. 10.1007/978-1-4939-3578-9_5 27008011 PMC4944384

[31] Norfo R, Zini R, Pennucci V, Bianchi E, Salati S, Guglielmelli P, Bogani C, Fanelli T, Mannarelli C, Rosti V, Pietra D, Salmoiraghi S, Bisognin A, et al, and Associazione Italiana per la Ricerca sul Cancro Gruppo Italiano Malattie Mieloproliferative Investigators. miRNA-mRNA integrative analysis in primary myelofibrosis CD34+ cells: role of miR-155/JARID2 axis in abnormal megakaryopoiesis. Blood. 2014; 124:e21–32. 10.1182/blood-2013-12-544197 25097177 PMC4186546

[32] Vannucchi AM, Lasho TL, Guglielmelli P, Biamonte F, Pardanani A, Pereira A, Finke C, Score J, Gangat N, Mannarelli C, Ketterling RP, Rotunno G, Knudson RA, et al. Mutations and prognosis in primary myelofibrosis. Leukemia. 2013; 27:1861–9. 10.1038/leu.2013.119 23619563

[33] Davis S, Meltzer PS. GEOquery: a bridge between the Gene Expression Omnibus (GEO) and BioConductor. Bioinformatics. 2007; 23:1846–7. 10.1093/bioinformatics/btm254 17496320

[34] Fagone P, Mangano K, Pesce A, Portale TR, Puleo S, Nicoletti F. Emerging therapeutic targets for the treatment of hepatic fibrosis. Drug Discov Today. 2016; 21:369–75. 10.1016/j.drudis.2015.10.015 26523773

[35] Smyth GK. Linear models and empirical bayes methods for assessing differential expression in microarray experiments. Stat Appl Genet Mol Biol. 2004; 3:Article3. 10.2202/1544-6115.1027 16646809

[36] Cheadle C, Vawter MP, Freed WJ, Becker KG. Analysis of microarray data using Z score transformation. J Mol Diagn. 2003; 5:73–81. 10.1016/S1525-1578(10)60455-2 12707371 PMC1907322

[37] Sanfilippo C, Castrogiovanni P, Imbesi R, Kazakowa M, Musumeci G, Blennow K, Zetterberg H, Di Rosa M. Sex difference in CHI3L1 expression levels in human brain aging and in Alzheimer’s disease. Brain Res. 2019; 1720:146305. 10.1016/j.brainres.2019.146305 31247206

[38] Torrisi F, Minafra L, Cammarata FP, Savoca G, Calvaruso M, Vicario N, Maccari L, Pérès EA, Özçelik H, Bernaudin M, Botta L, Russo G, Parenti R, Valable S. SRC Tyrosine Kinase Inhibitor and X-rays Combined Effect on Glioblastoma Cell Lines. Int J Mol Sci. 2020; 21:3917. 10.3390/ijms21113917 32486205 PMC7312922

[39] Kalluri R, Zeisberg M. Fibroblasts in cancer. Nat Rev Cancer. 2006; 6:392–401. 10.1038/nrc1877 16572188

[40] Schmitt A, Jouault H, Guichard J, Wendling F, Drouin A, Cramer EM. Pathologic interaction between megakaryocytes and polymorphonuclear leukocytes in myelofibrosis. Blood. 2000; 96:1342–7. 10.1182/blood.V96.4.1342 10942376

[41] Ren JJ, Huang TJ, Zhang QQ, Zhang HY, Guo XH, Fan HQ, Li RK, Liu LX. Insulin-like growth factor binding protein related protein 1 knockdown attenuates hepatic fibrosis via the regulation of MMPs/TIMPs in mice. Hepatobiliary Pancreat Dis Int. 2019; 18:38–47. 10.1016/j.hbpd.2018.08.008 30243878

[42] Wang JC, Novetsky A, Chen C, Novetsky AD. Plasma matrix metalloproteinase and tissue inhibitor of metalloproteinase in patients with agnogenic myeloid metaplasia or idiopathic primary myelofibrosis. Br J Haematol. 2002; 119:709–12. 10.1046/j.1365-2141.2002.03874.x 12437648

[43] Jensen MK, Holten-Andersen MN, Riisbro R, de Nully Brown P, Larsen MB, Kjeldsen L, Heickendorff L, Brünner N, Hasselbalch HC. Elevated plasma levels of TIMP-1 correlate with plasma suPAR/uPA in patients with chronic myeloproliferative disorders. Eur J Haematol. 2003; 71:377–84. 10.1034/j.1600-0609.2003.00096.x 14667201

[44] Gangat N, Tefferi A. Myelofibrosis biology and contemporary management. Br J Haematol. 2020; 191:152–70. 10.1111/bjh.16576 32196650

[45] Spampinato M, Giallongo C, Romano A, Longhitano L, La Spina E, Avola R, Scandura G, Dulcamare I, Bramanti V, Di Rosa M, Vicario N, Parenti R, Li Volti G, et al. Focus on Osteosclerotic Progression in Primary Myelofibrosis. Biomolecules. 2021; 11:122. 10.3390/biom11010122 33477816 PMC7832894

[46] Wang S, Hu M, Qian Y, Jiang Z, Shen L, Fu L, Hu Y. CHI3L1 in the pathophysiology and diagnosis of liver diseases. Biomed Pharmacother. 2020; 131:110680. 10.1016/j.biopha.2020.110680 32861071

[47] Tibullo D, Di Rosa M, Giallongo C, La Cava P, Parrinello NL, Romano A, Conticello C, Brundo MV, Saccone S, Malaguarnera L, Di Raimondo F. Bortezomib modulates CHIT1 and YKL40 in monocyte-derived osteoclast and in myeloma cells. Front Pharmacol. 2015; 6:226. 10.3389/fphar.2015.00226 26528182 PMC4604315

[48] Zhao T, Su Z, Li Y, Zhang X, You Q. Chitinase-3 like-protein-1 function and its role in diseases. Signal Transduct Target Ther. 2020; 5:201. 10.1038/s41392-020-00303-7 32929074 PMC7490424

[49] Bjørn ME, Andersen CL, Jensen MK, Hasselbalch HC. Circulating YKL-40 in myelofibrosis a potential novel biomarker of disease activity and the inflammatory state. Eur J Haematol. 2014; 93:224–8. 10.1111/ejh.12332 24689875

[50] Bhagwat N, Keller MD, Rampal RK, Shank K, de Stanchina E, Rose K, Amakye D, Levine RL. Improved Efficacy Of Combination Of JAK2 and Hedgehog Inhibitors In Myelofibrosis. Blood. 2013; 122:666. 10.1182/blood.V122.21.666.66623794067

[51] Schneider RK, Mullally A, Dugourd A, Peisker F, Hoogenboezem R, Van Strien PM, Bindels EM, Heckl D, Büsche G, Fleck D, Müller-Newen G, Wongboonsin J, Ventura Ferreira M, et al. Gli1^+^ Mesenchymal Stromal Cells Are a Key Driver of Bone Marrow Fibrosis and an Important Cellular Therapeutic Target. Cell Stem Cell. 2017; 20:785–800.e8. 10.1016/j.stem.2017.03.008 28457748 PMC6485654

[52] Guo X, Zhang H, Zhang Q, Li X, Liu L. Screening for and validation of a hepatic fibrosis-related pathway induced by insulin-like growth factor-binding protein-related protein 1. Eur J Gastroenterol Hepatol. 2016; 28:762–72. 10.1097/MEG.0000000000000631 27097355

[53] Tibullo D, Longo A, Vicario N, Romano A, Barbato A, Di Rosa M, Barbagallo I, Anfuso CD, Lupo G, Gulino R, Parenti R, Li Volti GL, Palumbo GA, et al. Ixazomib Improves Bone Remodeling and Counteracts sonic Hedgehog signaling Inhibition Mediated by Myeloma Cells. Cancers (Basel). 2020; 12:323. 10.3390/cancers12020323 32019102 PMC7073172

[54] Sinha S, Chen JK. Purmorphamine activates the Hedgehog pathway by targeting Smoothened. Nat Chem Biol. 2006; 2:29–30. 10.1038/nchembio753 16408088

[55] Wang X, Zhang W, Tripodi J, Lu M, Xu M, Najfeld V, Li Y, Hoffman R. Sequential treatment of CD34+ cells from patients with primary myelofibrosis with chromatin-modifying agents eliminate JAK2V617F-positive NOD/SCID marrow repopulating cells. Blood. 2010; 116:5972–82. 10.1182/blood-2010-02-269696 20858855 PMC3031385

[56] Tomchuck SL, Zwezdaryk KJ, Coffelt SB, Waterman RS, Danka ES, Scandurro AB. Toll-like receptors on human mesenchymal stem cells drive their migration and immunomodulating responses. Stem Cells. 2008; 26:99–107. 10.1634/stemcells.2007-0563 17916800 PMC2757778

[57] Giallongo C, Tibullo D, Camiolo G, Parrinello NL, Romano A, Puglisi F, Barbato A, Conticello C, Lupo G, Anfuso CD, Lazzarino G, Li Volti G, Palumbo GA, Di Raimondo F. TLR4 signaling drives mesenchymal stromal cells commitment to promote tumor microenvironment transformation in multiple myeloma. Cell Death Dis. 2019; 10:704. 10.1038/s41419-019-1959-5 31541083 PMC6754430

[58] Bhattacharyya S, Wang W, Qin W, Cheng K, Coulup S, Chavez S, Jiang S, Raparia K, De Almeida LM, Stehlik C, Tamaki Z, Yin H, Varga J. TLR4-dependent fibroblast activation drives persistent organ fibrosis in skin and lung. JCI Insight. 2018; 3:e98850. 10.1172/jci.insight.98850 29997297 PMC6124522

[59] Bellini MJ, Hereñú CB, Goya RG, Garcia-Segura LM. Insulin-like growth factor-I gene delivery to astrocytes reduces their inflammatory response to lipopolysaccharide. J Neuroinflammation. 2011; 8:21. 10.1186/1742-2094-8-21 21371294 PMC3056784

[60] Ngo VN, Young RM, Schmitz R, Jhavar S, Xiao W, Lim KH, Kohlhammer H, Xu W, Yang Y, Zhao H, Shaffer AL, Romesser P, Wright G, et al. Oncogenically active MYD88 mutations in human lymphoma. Nature. 2011; 470:115–9. 10.1038/nature09671 21179087 PMC5024568

[61] Gao B, Seki E, Brenner DA, Friedman S, Cohen JI, Nagy L, Szabo G, Zakhari S. Innate immunity in alcoholic liver disease. Am J Physiol Gastrointest Liver Physiol. 2011; 300:G516–25. 10.1152/ajpgi.00537.2010 21252049 PMC3774265

[62] Jiao S, Guan J, Chen M, Wang W, Li C, Wang Y, Cheng Y, Zhou Z. Targeting IRF3 as a YAP agonist therapy against gastric cancer. J Exp Med. 2018; 215:699–718. 10.1084/jem.20171116 29339449 PMC5789414

[63] Ege N, Dowbaj AM, Jiang M, Howell M, Hooper S, Foster C, Jenkins RP, Sahai E. Quantitative Analysis Reveals that Actin and Src-Family Kinases Regulate Nuclear YAP1 and Its Export. Cell Syst. 2018; 6:692–708.e13. 10.1016/j.cels.2018.05.006 29909276 PMC6035388

[64] Kim CL, Choi SH, Mo JS. Role of the Hippo Pathway in Fibrosis and Cancer. Cells. 2019; 8:468. 10.3390/cells8050468 31100975 PMC6562634

[65] Malara A, Gruppi C, Abbonante V, Cattaneo D, De Marco L, Massa M, Iurlo A, Gianelli U, Balduini CL, Tira ME, Muro AF, Chauhan AK, Rosti V, et al. EDA fibronectin-TLR4 axis sustains megakaryocyte expansion and inflammation in bone marrow fibrosis. J Exp Med. 2019; 216:587–604. 10.1084/jem.20181074 30733282 PMC6400533

[66] Martinaud C, Desterke C, Konopacki J, Pieri L, Torossian F, Golub R, Schmutz S, Anginot A, Guerton B, Rochet N, Albanese P, Henault E, Pierre-Louis O, et al. Osteogenic Potential of Mesenchymal Stromal Cells Contributes to Primary Myelofibrosis. Cancer Res. 2015; 75:4753–65. 10.1158/0008-5472.CAN-14-3696 26404004

